# Cerebral hemodynamics evaluation of FLAIR vascular hyperintensity in TIA patients with large artery severe stenosis or occlusion

**DOI:** 10.3389/fneur.2025.1589198

**Published:** 2025-05-14

**Authors:** Lichuan Zeng, Jiamei Wang, Qu Wang, Yaodan Zhang, Huaqiang Liao, Wenbin Wu

**Affiliations:** ^1^Hospital of Chengdu University of Traditional Chinese Medicine, Chengdu, China; ^2^Deyang Hospital Affiliated Hospital of Chengdu University of Traditional Chinese Medicine, Deyang, China

**Keywords:** arterial spin labeling, post label delay, FLAIR vascular hyperintensity, hyperintense vessel, magnetic resonance imaging

## Abstract

**Purpose:**

To assess the practicality and utility of employing dual post-label delay (PLD) arterial spin labeling (ASL) in transient ischemic attack (TIA) individuals exhibiting Fluid-attenuated inversion recovery (FLAIR) vascular hyperintensity (FVH).

**Materials and methods:**

We conducted a retrospective review of clinical data from TIA patients presenting with unilateral severe atherosclerotic stenosis or obstruction of either the intracranial internal carotid artery or the middle cerebral artery. Participants were categorized into two groups based on the presence or absence of FVH: FVH positive and FVH negative. All individuals underwent pseudo-continuous ASL perfusion imaging, utilizing distinct PLD durations (1,525 and 2,525 ms) alongside qualitative visual assessments of ASL perfusion irregularities. Standardized TIA evaluations, which included medical history reviews, neuropsychological assessments, and ABCD2 scoring, were performed on all subjects. We explored the correlations between FVHs, clinical manifestations, vascular risk factors, and perfusion metrics.

**Results:**

A total of 50 patients were included in this investigation, with FVH detected in 16 subjects (32.0%). The ABCD2 score was notably elevated within the FVH positive cohort compared to the FVH negative group. At a PLD of 1,525 ms, cerebral blood flow (CBF) values for the affected and healthy hemispheres in the FVH positive group were recorded at 19.55 ± 6.67 and 40.32 ± 6.83, respectively; corresponding values in the FVH negative group were 23.74 ± 5.03 and 46.43 ± 7.91. For a PLD of 2,525 ms, the CBF values for the affected and healthy sides in the FVH positive group were 34.11 ± 5.87 and 50.27 ± 8.57, while the FVH negative group recorded values of 42.79 ± 7.03 and 52.07 ± 7.29, respectively. The differential CBF (ΔCBF) for the affected side in the FVH positive and negative groups was 14.57 ± 4.34 and 19.05 ± 6.10, respectively. A significant negative correlation was established between ΔCBF and ABCD2 scores (Kendall’s tau-b = −0.578, *p* < 0.001).

**Conclusion:**

The findings of this study indicate a strong association between the presence of FVH signs and a marked reduction in cerebral blood flow, as well as diminished blood flow reserve. This underscores the potential role of FVH as a biomarker for hemodynamic impairment in TIA patients.

## 1 Background

Cerebral stenotic or occlusive disorders are associated with a significant decrease in flow velocity attributable to vascular stenosis or occlusion, and the development of collateral blood flow (1). Patients suffering from atherosclerotic steno-occlusive cerebrovascular disease exhibit a considerable, albeit variable, risk of subsequent stroke. A transient ischemic attack (TIA) is typically defined by the rapid onset of a focal neurological deficit of vascular origin, which completely resolves within 24 h (2–4). TIA patients with steno-occlusive cerebrovascular conditions have long been acknowledged as being at heightened risk for subsequent stroke events.

Fluid-attenuated inversion recovery (FLAIR) vascular hyperintensities (FVHs) are described as focal, linear, or serpentine hyperintensities that correspond to arteries within the subarachnoid space and are frequently observed in patients with large artery severe stenosis or occlusion (LASO) (5, 6). FVHs are often detected in individuals with acute ischemic stroke, suggesting considerable hemodynamic impairment and sluggish retrograde flow in the ischemic region (7, 8). Possible explanations for their presence include stagnant blood flow and delayed antegrade or retrograde filling (9). The collateral circulation of the leptomeninges plays a critical role in certain clinical scenarios involving transient ischemic attack (TIA) patients, particularly those with LASO. Some individuals within this cohort exhibit FLAIR vascular hyperintensity, while others do not. However, investigations focusing on the correlation between focal vascular hyperintensities (FVHs) and cerebral perfusion remain sparse.

The use of dynamic magnetic resonance imaging with sensitivity to contrast agents may elevate the potential for complications related to the administration of exogenous contrast materials (10, 11). Arterial spin labeling (ASL) magnetic resonance perfusion imaging serves as a valuable technique for visualizing cerebral perfusion and assessing cerebral blood flow (CBF). This method employs magnetically tagged protons in arterial blood as an intrinsic tracer, thereby negating the necessity for external contrast agents or radioactive tracers (12–16). A strong correlation has been consistently identified in many studies when assessing cerebral perfusion using ASL and CT perfusion (17, 18). An essential parameter in ASL is the post-label delay (PLD) time, defined as the interval between the conclusion of the pulse sequence and the subsequent image acquisition. A brief PLD may not allow for adequate delivery of labeled blood to the target tissue, while an excessively long PLD can result in substantial T1 decay, ultimately diminishing the signal-to-noise ratio (19, 20). When utilizing a singular conventional PLD, the labeled bolus may not completely reach the parenchyma intended for examination, particularly in junctional zones, leading to significant local signal attenuation that could be misinterpreted as false hypoperfusion. Conversely, in patients with well-developed collateral circulation, a single conventional PLD may present apparent hyperperfusion in areas where collateral blood flow is stagnant, a phenomenon referred to as the arterial transit artifact (19). It is thus recommended to employ multiple PLD strategies to enhance the accuracy of CBF quantification (21, 22). The aim of this study was to assess the cerebral hemodynamic state in TIA patients experiencing severe stenosis or occlusion of large arteries, particularly those exhibiting FLAIR vascular hyperintensity.

## 2 Materials and methods

### 2.1 Subjects

We performed a retrospective analysis of TIA patients diagnosed with unilateral severe atherosclerotic stenosis (greater than 70%) or occlusion of the intracranial internal carotid artery (ICA, C6, or C7 segment) or middle cerebral artery (MCA, M1 segment) at our institution from January 2023 to December 2024. The inclusion criteria encompassed: (1) Transient neurological symptoms that a clinical neurologist assessed to potentially have a vascular origin; (2) Confirmation of unilateral stenosis or occlusion of the ICA or MCA via MRA or CTA; (3) Non-specific findings on general MRI and diffusion-weighted imaging (DWI); and (4) Completion of an MRI study incorporating ASL with PLD values of 1,525 and 2,525 ms. Exclusion criteria included: (1) Presence of intracranial hemorrhage, brain tumors, cranial trauma, psychiatric disorders, or other recognized brain abnormalities; (2) Poor quality of ASL imaging and failure to perform standard imaging; (3) Other cerebrovascular conditions such as Moyamoya disease or various cerebrovascular malformations; and (4) Incomplete or absent clinical data for patients. All subjects underwent routine screening for TIA, with ABCD2 scores evaluated by trained neurologists through the review of electronic medical records. The study received approval from the Ethics Committee of the Hospital of Chengdu University of Traditional Chinese Medicine, which waived the necessity for written informed consent due to the retrospective nature of the research.

### 2.2 MR imaging

All patients underwent MRI scans utilizing a Discovery MR750 3.0 T system (GE Healthcare, Milwaukee, WI, United States) outfitted with an 8-channel phased array head coil. The imaging protocol included T1-weighted imaging (T1WI), T2-weighted imaging (T2WI), T2 fluid-attenuated inversion recovery (T2-FLAIR), diffusion-weighted imaging (DWI), angiography (MRA) and pseudo-continuous arterial spin labeling (ASL) perfusion imaging were performed using two distinct post-labeling delays (PLDs) of 1,525 and 2,525 ms. The acquisition of the whole-brain three-dimensional pCASL perfusion sequence was executed utilizing a fast spin-echo methodology with background suppression, adhering to the specified parameters: labeling duration of 1,525 ms, repetition time (TR) of 4,632 ms, echo time (TE) of 10.5 ms, 36 slices, a slice thickness of 4.0 mm, a field of view of 24 cm × 24 cm, and an acquisition duration of 4 min and 29 s. Subsequently, the PLD was adjusted to 2,525 ms, while retaining all other parameters constant, resulting in an acquisition time of 5 min and 9 s.

### 2.3 Image evaluation

In terms of image evaluation, focal FVH was characterized as a serpentine or speckled hyperintensity located in the sulcus and subarachnoid space on T2-FLAIR imaging. Two independent neuroradiologists, each possessing a minimum of 5 years of MRI experience, scrutinized the images to identify the presence of FVH signs and ASL perfusion anomalies through qualitative visual assessment, deliberately excluding identifiable and clinical information. The ASL cerebral blood flow (CBF) maps for both patient groups underwent post-processing and were generated utilizing the Function Tool (Advanced Workstation 4.6; GE Healthcare). For each patient, a rounded region of interest (ROI) was meticulously delineated on the 3D PCASL images to symmetrically assess CBF values on both the affected and healthy hemispheres. Careful placement of the ROIs was ensured to circumvent blood vessels, cerebral sulci, or cerebral cisterns. Three ROIs were employed in each brain region to quantify CBF, and the average value was utilized for subsequent analysis. In patients experiencing hemispheric transient ischemic attacks, the assessment of perfusion disturbances was conducted to ascertain their correlation with the hemispheric localization of presenting symptoms.

### 2.4 Statistical analysis

Inter-observer agreement was assessed using kappa (*κ*) statistics. κ > 0.6 is considered to indicate good agreement, while κ > 0.8 is regarded as excellent. Continuous variables that follow a normal distribution are presented as mean ± standard deviation (SD), whereas categorical variables are reported in terms of frequency (%). The differences in each of the categorical variables between the two groups were analyzed using one-way ANOVA, the chi-square test, and Fisher’s exact test (when the expected cell frequency was < 5). Statistical significance was set at *p* < 0.05. All statistical analyses were performed using SPSS software (version 20.0, SPSS Inc., Chicago, IL, United States).

## 3 Results

### 3.1 Demographic and clinical information

Fifty patients (mean age: 60.5 ± 9.0 years; 27 males) satisfied the inclusion criteria and were recruited for the study. The inter-reader agreement for FVH detection was determined to be good (*κ* = 0.86). Among the cohort, FVH was identified in 16 patients (32.0%). The ABCD2 scores for subjects in the study were 4 (3–5) in the FVH-positive group and 2 (1–3) in the FVH-negative group, showcasing a statistically significant difference (*p* < 0.05). Furthermore, significant disparities were noted between the FVH negative and FVH positive groups in terms of prior stroke history and symptom duration (*p* < 0.05). Notably, cardiovascular risk factors, including hypertension, hyperlipidemia, and smoking history, did not exhibit significant differences between patients with and without FVH. Detailed characteristics of TIA patients, both with and without FVH signs, are outlined in Table 1.

**Table 1:** Baseline characteristics of the TIA patients with and without FVH sign.

	FVH(+) (*n* = 16)	FVH(−) (*n* = 34)	*p*-value
Age (years), mean ± SD	64.0 ± 6.5	58.9 ± 9.6	0.061
Male gender	10(62.5%)	17(50.0%)	0.408
Previous stroke	5(31.3%)	2(2.9%)	0.016*
ABCD2 score (median, IQR)	4(3–5)	2(1–3)	<0.001*
Hypertension	11(68.8%)	19(55.9%)	0.386
Diabetes mellitus	4(25.0%)	8(23.5%)	0.910
Hyperlipidemia	7(43.8%)	8(23.5%)	0.146
Smoking history	7(43.8%)	12(35.3%)	0.472
Atrial fbrillation	4(25.0%)	8(23.5%)	0.910
Symptom duration (>1 h)	13(81.3%)	15(44.1%)	0.014*
Coronary heart disease	7(43.8%)	8(23.5%)	0.146
Occlusive site			
MCA	6(37.5%)	9(26.5%)	0.427
ICA	10(62.5%)	25(73.5%)	0.427

ABCD2, age, blood pressure, clinical symptoms, duration of TIA and diabetes; FVH, FLAIR vascular hyperintensity; MCA, middle cerebral artery; ICA, internal carotid artery. *There is significant difference between two groups.

### 3.2 Characteristics of cerebral perfusion parameters

**Figure 1:**
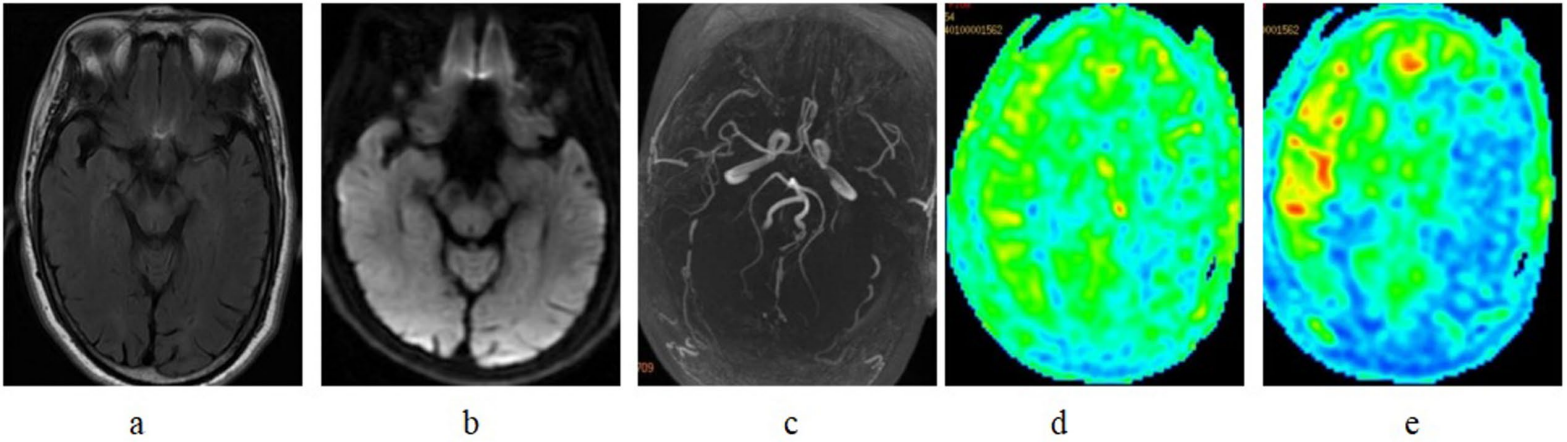
FLAIR vascular hyperintensity in a patient with left middle cerebral artery occlusion. **(a)** Serpentine hyperintense signal of the middle cerebral artery branches in the left sylvian fissure in T2 FLAIR. **(b)** DWI demonstrate negative finding. **(c)** MRA showed occlusion of the left MCA M1. **(d)** On ASL with a PLD of 1525 ms markedly decreased ASL signals are noted in the extended area of the left hemisphere. ASL with a PLD of 2525 ms, **(e)** the decreased area is somewhat improved, but there is still a slight laterality compared with the right side.

**Figure 2:**
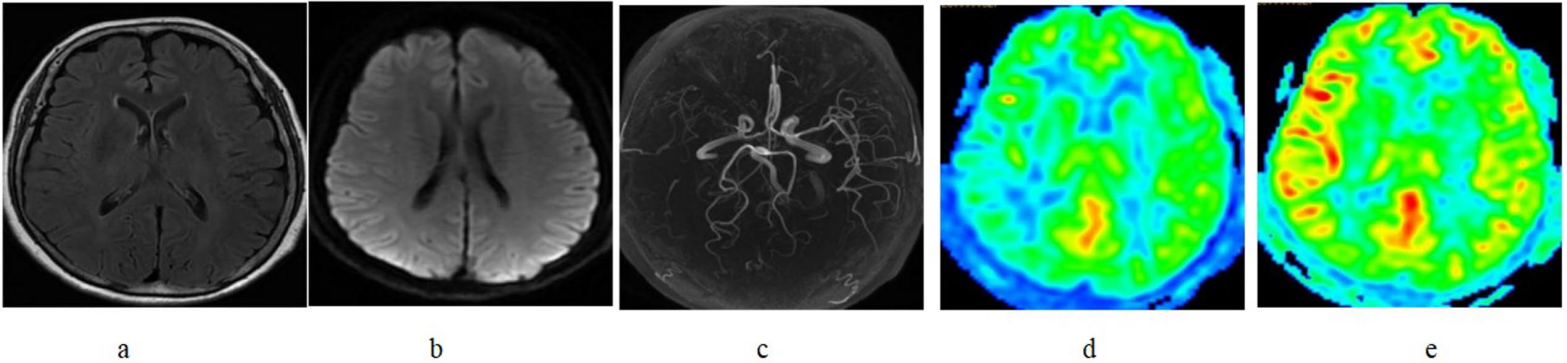
FLAIR vascular hyperintensity absent in a patient with right middle cerebral artery occlusion. **(a)** FLAIR vascular hyperintensity absent in T2 FLAIR and **(b)** DWI demonstrate negative finding. **(c)** MRA showed occlusion of the right MCA M1. The area of the right MCA demonstrated to be hypoperfused on ASLwith a PLD of 1525 ms **(d)** but improved on ASL with a PLD of 2525 ms **(e)**.

The ASL markers in the current study demonstrated good reproducibility between raters (*κ* = 0.82). The detailed ASL perfusion data for patients from both groups is summarized in Table 2. For the PLD of 1,525 ms, the CBF values were recorded at 19.55 ± 6.67 and 40.32 ± 6.83 for the affected and healthy sides, respectively, within the FVH positive group, whereas the FVH negative group exhibited corresponding values of 23.74 ± 5.03 and 46.43 ± 7.91. In the scenario of a PLD of 2,525 ms, the CBF values were noted as 34.11 ± 5.87 and 50.27 ± 8.57 for the affected and healthy sides in the FVH positive group, whereas in the FVH negative group, the respective values were 42.79 ± 7.03 and 52.07 ± 7.29. The D-value (ΔCBF) for both groups was compared on the affected side across different PLDs, yielding values of 14.57 ± 4.34 and 19.05 ± 6.10, respectively. Representative cases of FVH positive and FVH negative patients with varying PLDs are depicted in Figures 1, 2. Notably, a significant negative correlation was identified between ΔCBF and ABCD2 scores (Kendall’s tau-b = −0.578, *p* < 0.001).

**Table 2:** Results of ASL in different PLDs in patients with and without FVH sign.

CBF (mL·100 g^−1^·min^−1^)	FVH(+) (*n* = 16)		FVH(−) (*n* = 34)		*p*-value
	Affected side	Healthy side	Affected side	Healthy side	
CBF1	19.55 ± 6.67	40.32 ± 6.83	23.74 ± 5.03	46.43 ± 7.91	0.017*
CBF2	34.11 ± 5.87	50.27 ± 8.57	42.79 ± 7.03	52.07 ± 7.29	<0.001*
△CBF	14.57 ± 4.34		19.05 ± 6.10		0.011*

CBF1, CBF in PLD 1,525 ms; CBF2, CBF in PLD 2,525 ms; △CBF, CBF2-CBF1. *There is significant difference in affected side between two groups.

## 4 Discussion

This investigation focused on the correlation between FVH and perfusion through ASL at varying PLDs in patients suffering from TIA and intracranial aortic stenosis or occlusion. The study presents three primary conclusions. Firstly, FVHs were identified in 32% of TIA patients who underwent LASO, aligning with findings from prior research (23). Secondly, our analysis indicated that TIA patients with LASO exhibiting positive FVH were substantially more likely to have reduced CBF and a more critically compromised blood flow reserve compared to those without FVH. Correlation assessments demonstrated significant negative relationships between ΔCBF and ABCD2 scores. Thirdly, patients with TIA and positive FVH encountered a notably elevated ABCD2 risk score, a frequently utilized metric for assessing stroke risk post-TIA. These results provide valuable insights for clinicians in understanding hemodynamic conditions and collateral compensation, which are essential for accurately identifying disease states and presenting reliable imaging evidence for both primary and secondary stroke prevention.

While much of the existing literature has concentrated on FVH manifestations in patients with acute cerebral infarctions, the prevalence of FVH is highly variable across different studies. The assessment of FVH in TIA patients remains less comprehensive compared to those with ischemic strokes. Maeda et al. (24) evaluated the progression of FVH in acute and subacute cerebral infarctions within the middle cerebral artery territory and observed that FVH appeared in 100% of evaluations conducted within 24 h of symptom onset, but only in 18% of assessments performed 5–9 days post-symptom onset. The occurrence of FVH correlates with the duration between stroke onset and MRI imaging, with its frequency diminishing over time. Ding et al. (25) noted that FVH was detected in as many as 81.6% (31/38) of hospitalized TIA patients exhibiting severe stenosis or occlusion, with MR scans executed within 48 h of symptom onset, a timeframe significantly shorter than that of our study.

Most research suggests a relationship between FVH and either large-vessel occlusion or severe stenosis, as well as hemodynamic impairment. Lyu et al. (5) examined whether FVH could serve as a prognostic indicator for ischemic events in patients with ICA or MCA occlusion. Their findings indicated that the FVH-ASPECTS was substantially lower in the asymptomatic occlusion cohort compared to the symptomatic group, implying that FVH might be predictive of stroke occurrence. Bunker and Hillis (26) reported that the typical location of FVHs matched regions of hypoperfusion in corresponding vascular territories captured via perfusion-weighted imaging. Nam et al. (27) investigated the relationship between FVH and early ischemic lesion recurrence in patients with lesion-negative TIA. Their results demonstrated that FVH is significantly associated with early ischemic lesion recurrence in this patient population. Our study quantitatively illustrates that TIA patients with significant arterial vessel occlusion who also present with FVH exhibit markedly lower CBF values and more severely diminished blood flow reserves than those without FVH. In cases of unilateral LASO, it is essential to consider the effects of delayed anterograde flow along with both primary and secondary collateral circulations when evaluating hemodynamic status.

Disruption of the blood–brain barrier (BBB) represents a critical pathological feature of ischemic stroke. Preserving BBB integrity is vital for maintaining central nervous system homeostasis (28). Cerebrovascular stenosis triggers pathological cascades via hemodynamic alterations. Atherosclerotic narrowing not only decreases cerebral perfusion pressure but also elevates turbulent flow, thereby inducing endothelial shear stress. This stress activates matrix metalloproteinases (MMPs) and compromises tight junction proteins, leading to BBB dysfunction. The resultant BBB compromise facilitates erythrocyte extravasation and hemoglobin degradation, releasing ferrous iron that catalyzes Fenton reactions. The subsequent production of reactive oxygen species (ROS) perpetuates neuroinflammation and exacerbates BBB disruption through vascular endothelial growth factor (VEGF)-mediated vascular remodeling. Importantly, hemosiderin deposition in perivascular spaces generates paramagnetic susceptibility effects detectable on susceptibility-weighted imaging (SWI) MRI. Furthermore, chronic iron overload accelerates tau phosphorylation and amyloid-*β* aggregation via ferroptosis pathways, establishing a self-sustaining cycle of neurodegeneration (29). Uchida et al. (30) conducted a combined quantitative MRI analysis using QSM and R2* relaxometry, demonstrating that increased iron concentration in ischemic lesions is associated with reduced improvement in neurological outcomes following stroke rehabilitation.

Conventional magnetic resonance imaging has a satisfactory resolution ratio for components of brain tissue, but cannot provide blood flow perfusion information. ASL imaging enables detection of absolute perfusion values. PLD is the most important parameter contributing to the accurate assessment of CBF (31, 32). Cerebral blood flow imaging can sensitively and specifically detect abnormal perfusion changes (33). The present study targeted the noninvasive assessment of cerebral hemodynamics with a quantitative measure of CBF perfusion using the ASL technique. This technique has good reproducibility and does not require radiation or gadolinium-based tracers, thereby avoiding potential adverse effects. CBF readings obtained from a single PLD scan may not accurately reflect the true cerebral perfusion status, particularly in patients with extensive vascular lesions. A PLD of 2,525 ms may detect slower blood flow through secondary collateral circulation, while a PLD of 1,525 ms may not capture such dynamics. This study validated dual-PLD settings of 1,525 and 2,525 ms for assessing the hemodynamic condition of patients with transient ischemic attack (TIA) who underwent large artery stenosis occlusion. Moreover, we explored the clinical significance of supplementary ASL and FVH signs. Patients experiencing transient ischemic attacks frequently exhibit similar pathophysiological mechanisms to individuals suffering from other cerebrovascular stenotic or obstructive diseases, characterized by a pronounced reduction in blood flow velocity resulting from arterial stenosis or occlusion, alongside the establishment of collateral circulation. Intracranial atherosclerosis is a notable contributor to cerebral stenosis and insufficient cerebral perfusion, playing a crucial role in both the initial onset and the recurrence of ischemic strokes (5). Individuals exhibiting transient neurological symptoms alongside confirmed evidence of LASO are at an increased risk of subsequent strokes due to compromised CBF and disturbed cerebral perfusion. The ABCD2 scoring system, which is based on specific risk factors and clinical presentations of TIA, revealed that patients with TIA and FLAIR hyperintensities (FVH) had significantly elevated ABCD2 risk scores, potentially correlating with adverse outcomes. Collateral circulation can sustain brain tissue viability for extended periods following the occlusion of major cerebral arteries, thereby serving a vital function in TIA patients. Therefore, enhancing or preserving collateral circulation emerges as a promising therapeutic target. Given that chronic hemodynamic impairment often leads to progressive cortical neuronal degeneration, early intervention is advocated for TIA patients presenting with FVH to avert stroke occurrence.

The application of ASL magnetic resonance imaging with dual PLD holds significant potential for evaluating cerebral hemodynamics in TIA patients. This method addresses limitations of single-PLD ASL by capturing both early and delayed perfusion phases, which is critical for assessing collateral-dependent blood flow and cerebrovascular reserve (CVR) in regions with prolonged arterial transit times (ATT). For instance, studies in patients with internal carotid artery steno-occlusion demonstrated that hypoperfusion observed at PLD 1.5 s often improved at PLD 2.5 s due to delayed collateral flow, as validated by digital subtraction angiography (DSA) (19, 34). This dual-PLD approach also differentiates stagnant collateral pathways from functional hyperperfusion, which is essential for identifying tissue at risk of ischemic injury in TIA patients (19). Furthermore, dual-PLD ASL correlates with acetazolamide-challenged SPECT in assessing CVR, highlighting its utility for noninvasive evaluation of hemodynamic compromise (35). In periictal hyperperfusion studies, dual PLD revealed distinct hemodynamic patterns (“fast flow” vs. “gradual flow”), suggesting its adaptability to dynamic perfusion changes (36). For TIA patients, combining these capabilities could enable precise stratification of cerebral hemodynamic status, guiding interventions to prevent stroke progression. The method’s repeatability and lack of contrast agents further support its practicality in serial monitoring of TIA-related perfusion alterations (34, 35).

Despite meticulous participant selection and data scrutiny, certain limitations must be acknowledged. Firstly, this investigation was a retrospective study conducted at a single center, which might introduce selection bias. Further prospective research is warranted to elucidate the underlying pathophysiological mechanisms. Secondly, while the study cohort was relatively small, it encompassed a homogenous group of TIA patients who underwent dual PLD assessments. Future research should aim to replicate this investigation with a larger sample size. Thirdly, longitudinal studies are essential to ascertain the prognostic significance of FVH.

## 5 Conclusion

In conclusion, this study establishes that FVH signs exhibit a strong correlation with diminished cerebral blood flow and a significantly impaired blood flow reserve, potentially reflecting the underlying pathomechanisms associated with stroke. The dual-PLD approach is a noninvasive and straightforward method for evaluating cerebral hemodynamics in TIA patients. We are optimistic that this methodology will evolve into a valuable instrument for prevention and early intervention in the future.

## Data Availability

The raw data supporting the conclusions of this article will be made available by the authors, without undue reservation.
